# Serum MMP7, MMP10 and MMP12 level as negative prognostic markers in colon cancer patients

**DOI:** 10.1186/s12885-016-2515-7

**Published:** 2016-07-18

**Authors:** Fee Klupp, Lena Neumann, Christoph Kahlert, Johannes Diers, Niels Halama, Clemens Franz, Thomas Schmidt, Moritz Koch, Juergen Weitz, Martin Schneider, Alexis Ulrich

**Affiliations:** Department of General, Visceral and Transplantation Surgery, University of Heidelberg, Im Neuenheimer Feld 110, 69120 Heidelberg, Germany; National Center for Tumor diseases, Medical Oncology and Internal medicine VI, Tissue Imaging and Analysis Center, Bioquant, University of Heidelberg, Im Neuenheimer Feld 267, 69120 Heidelberg, Germany; Department of Visceral, Thoracic and Vascular Surgery, University of Dresden, Fetscherstr. 74, 01307 Dresden, Germany

**Keywords:** Colon cancer, Serum, MMP, Survival, Prognostic marker

## Abstract

**Background:**

Matrixmetalloproteinases (MMPs) comprise a family of zinc-dependent endopeptidases which are involved in angiogenesis, tumor invasion and metastatic formation. Up to date, the prognostic relevance of MMPs in serum of patients with colon cancer remains unknown. Thus, we wanted to assess an expression pattern of MMPs in a homogenous cohort of colon cancer patients to assess their potential as prognostic biomarkers.

**Methods:**

Differences in the expression pattern of MMP7, MMP10 and MMP12 in 78 serum specimens of patients with an adenocarcinoma of the colon and serum specimens of a healthy control group were assessed using Luminex-100 technologies. Subsequently, we correlated these results with histopathological and clinical data of the patients.

**Results:**

Luminex based expression analysis revealed a significant overexpression of MMP7 and an overexpression of MMP10 and MMP12 in the sera of colon cancer patients compared to the healthy control group. Patients with vascular invasion showed a significantly higher MMP12 expression than V0-staged patients. Moreover overexpression of MMP7, MMP10 and MMP12 in colon cancer patients´ sera displayed a significantly impaired overall survival. Multivariate analysis revealed high MMP10 serum levels to be an independent adverse prognostic marker in colon cancer patients.

**Conclusions:**

Expression patterns of MMP7, MMP10 and MMP12 in colon cancer patients´ sera are different compared to serum specimens of healthy individuals. Furthermore, overexpression of MMP7, MMP10 and MMP12 in colon cancer patients´ sera correlates with a dismal prognosis and may help to stratify patients into different risk groups.

**Electronic supplementary material:**

The online version of this article (doi:10.1186/s12885-016-2515-7) contains supplementary material, which is available to authorized users.

## Background

Colorectal cancer (CRC) is one of the most prevalent cancer entities and the second leading cause of cancer-related death in the western world [1, 2]. For all stages of CRC the 5-year survival rate is about 60 % [3]. Survival depends strongly on the histological stage with favorable survival rates in stage I and II whereas the outcome drastically decreases with lymph node positivity or distant metastases [4]. Adjuvant chemotherapy in advanced stages improves survival rates heavily, but with the restrictions of general cytotoxity [5, 6]. It is known that a percentage of patients is either under- respectively overtreated due to the decision of adjuvant chemotherapy referring current clinical guidelines [7]. Therefore individualized and tailored therapeutic strategies have turned into focus. Hence other predictive markers then eg. Dukes stage which influence tumor progression and survival were investigated like - to name only few - microsatellite instability or cyclooxygenase-2 expression with the aim to proceed a step towards new therapeutic strategies additionally to chemo- and/or radiotherapy [8, 9]. Matrixmetalloproteinases (MMPs) comprise a family of at least 25 secreted and cell surface zinc-dependent endopeptidases involved in tumor progression, angiogenesis, invasion of surrounding tissue and metastatic formation and evasion of the immune system [10]. They are capable to degrade all components of the extracellular matrix and even non-matrix proteins, necessarily needed for tumor invasion and metastatic spread [11, 12]. According to their substrate specificity or additional structural domain they are divided into subgroups like Collagenases, Gelatinases, Stromelysins, Matrilysins and Membrane-type MMPs. Synthesized as inactive zymogens, they become functional by proteolytic removal of the propeptide prodomain [13]. The activity of MMPs is regulated by differential expression and post-translational processes. Activating factors include transcription factors such as LEF-1 (lymphoid enhancer binding factor-1) or ß-catenin, other MMPs, serin proteinases, cytokines and growth factors [10, 13]. Counterparts in terms of expressional inhibition are the tissue inhibitors of matrixmetalloproteinases (TIMPs), RECK (reversion-inducing cysteine-rich protein with Kazal motifs), α2-Macroglobulin or Thrombospondin [14–16].

Upregulation of several MMPs including MMP7, MMP10 and MMP12 in cancerous tissue and adverse association with survival has been evaluated likewise in colorectal cancer [17, 18]. Moreover secretion of MMPs into the bloodstream is proposed [19]. However prognostic impact of MMP expression in colorectal cancer patients´ sera has only been assessed partially. In this study we have chosen three MMPs namely MMP7, MMP10 and MMP12 for investigation. However, to our knowledge until now there is no study assessing serum MMP10 and MMP12 level in a homogenous collective of colon cancer patients’ sera in regard to overall survival. The prognostic impact of MMP7 serum level in advanced colorectal cancer has already been evaluated before [20]. Therefore assessment of serum MMP7 level serves as a validation for underlining the representative study collective of patients. Hence, we investigated multiplex bead-based immunoassay-technology measuring MMP7, MMP10 and MMP12 serum level in a homogenous collective of 78 patients suffering from colon cancer and a healthy control serum group to assess presumed differences in expression pattern and correlations with clinicopathological characteristics and survival.

## Methods

### Patients

Serum samples of 78 patients with primary adenocarcinoma of the colon were included in our current study. Patients received surgical treatment at the Department of General, Visceral, and Transplantation Surgery, University of Heidelberg, Germany between September 2007 and January 2012. Serum specimens of 38 healthy individuals without any known comorbidities served as control serum group. A written informed consent of all patients and healthy donors was obtained and the study was approved by the ethics committee of the University of Heidelberg. Clinical data included gender, date of birth, age at surgery, size, tumor location, histopathological diagnosis including TNM classification and UICC-stage, R-classification, grading, adjuvant chemotherapy, postoperative complications and overall survival (time from operation up to death or last follow up). Median follow up time was 901 ± 469 days (range: 6–1980 days).

### Tissue material and preparation

Patients´ sera were obtained during surgery and immediately stored at −80 °C. Likewise, healthy serum samples were immediately stored at −80 °C after removal. Total protein concentration was measured using Pierce® BCA Protein Assay Kit (Thermo Fisher Scientific Inc, Rockford, IL, USA) and Infinite 200® PRO Reader (Tecan Group Ltd., Männedorf, Switzerland) according to the manufacturer’s protocol. Magellan™ Data Analysis Software (Tecan Group Ltd., Männedorf, Switzerland) was taken for analysis of these data. All serum samples were diluted to a concentration of 0.1 μg/μl of total protein.

### Luminex based multiplex assay

Serum samples for the detection of MMP7, MMP10 and MMP12 were processed using Milliplex MAP Human MMP Assay Kits (Merck Millipore, Millipore Corporation, Billerica, MA, USA) according to the manufacturer’s protocol. The exact concentration of these markers were detected in each sample by Luminex 100™ reader (Luminex Corporation, Austin, Texas, USA).

### Statistics

Statistical analysis of the data and calculations were conducted with Excel 2010 (Microsoft Corporation, Redmond, WA) and SPSS version 21 (SPSS, IBM Corporation, Armonk, NY).

Wilcoxon-signed rank test was used to determine differences in the expression pattern. Expression bars were presented using mean concentration [pg/ml] + SEM. Kaplan-Meier method was employed to estimate cancer related survival. Differences between the survival curves were evaluated by log-rank test. For survival analysis Cox proportional hazards model was taken to calculate survival related hazard ratios. Multivariate analysis was performed using Cox proportional hazards regression including the following covariates: age, gender, UICC stage, TNM-classification, R-Stage, adjuvant chemotherapy, anastomotic leakage and relative serum expression of MMP7, MMP10 and MMP12 in colon cancer patients´ sera vs. sera of healthy control serum group. The comparison of clinical parameters and relative serum expression of MMPs was assessed using the Mann–Whitney-U-Test and the analysis of variance (Anova) with the post-hoc Tukey’s test. Contingency table analysis was performed using the Pearson’s chi-square test. Results were considered significant at a *p*-value less than 0.05.

## Results

### Patients’ characteristics

78 patients suffering from adenocarcinoma of the colon were included into the study (Table 1). Median age at the time of operation was 63 years. 28 patients died during follow-up. Mean overall survival of all patients was 44.2 months.

**Table 1:** Correlation of clinical parameters with overall survival; na = not available

Patient characteristics	Number of patients (*n* = 78)	Mean overall Survival (months)	*p*-value
Gender			0.37
Male	54	43.07	
Female	24	49.62	
Age at operation			0.514
<median = 64 years	37	43.82	
≥ median = 64 years	41	42.68	
Anastomotic leakage			0.841
Yes	7	50.10	
No	71	44.42	
T-Stage			0.495
T1	2	na	
T2	8	32.8	
T3	54	40.04	
T4	14	38.28	
N-Stage			0.08
N0	30	49.18	
N1	30	37.82	
N2	18	41.79	
M-Stage			0.01
M0	55	46.69	
M1	23	36.55	
Occurrence of liver metastases			0.002
Liver metastases (synchronous and metachronous)	26	35.53	
No liver metastases	52	51.51	
Resection margin status			0.121
R0	68	45.98	
R1	4	35.25	
Rx	6	29.02	
Grading			0.708
G1	0		
G2	54	43.55	
G3	23	45.97	
Data not available	1		
Adjuvant chemotherapy			0.083
Yes	47	40.66	
No	31	44.20	

Each patient underwent colon cancer surgery according to the localization of the tumor: 28 patients had right colectomy, 13 extended right colectomy, 1 had extended left colectomy, 9 left colectomy, 19 underwent sigmoidectomy, 6 patients underwent high anterior rectum resection, 2 patients had subtotal colectomy.

At the time of diagnosis 23 patients were classified M1, 55 patients as M0. Patients with a metastatic disease (M1) had a significantly shortened overall survival (*p* = 0.01). The mean overall survival for stage M0 patients was 46.7 months whereas it was 36.6 months for patients with distant metastases (Table 1).

### Expression of MMP7, MMP10 and MMP12 in colon cancer patients´ sera and sera of healthy individuals

The expression of MMP7, MMP10 and MMP12 were determined in serum samples of 78 colon cancer patients and 38 serum samples of healthy individuals using Luminex 100™ technology. Case numbers vary between different MMPs due to technical measurement procedures.

**Fig. 1:**
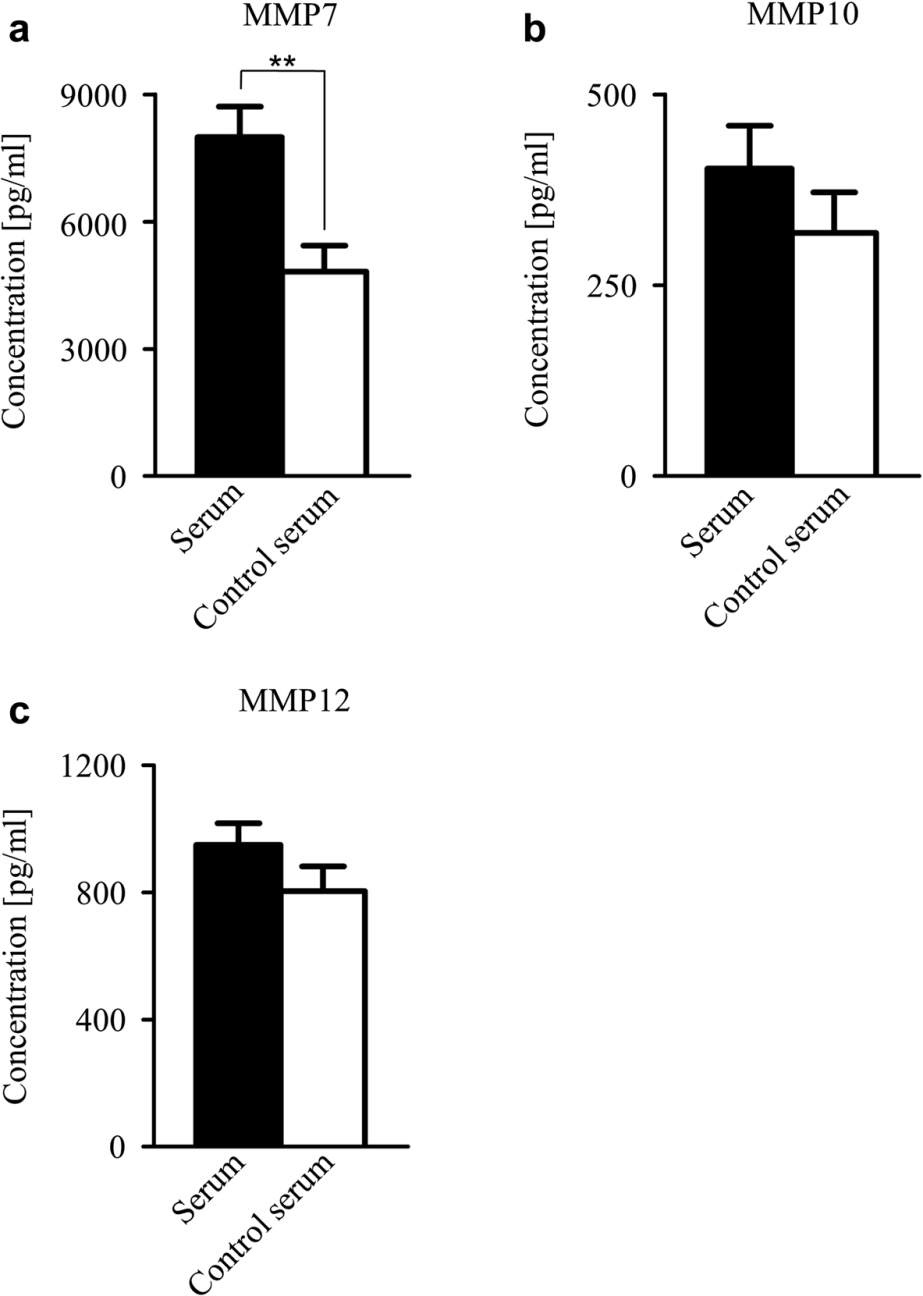
Expression analysis of MMP7, MMP10 and MMP12 in colon cancer patients´ sera and sera of healthy individuals. Expression of (**a**) MMP7 (*n* = 76 respectively 37 patients) (*p* = 0.003) was significantly underexpressed, expression of (**b**) MMP10 (*n* = 76 respectively 38 patients) and (**c**) MMP12 (*n* = 73 respectively 32 patients) was reduced in colon cancer patients´ sera as compared to control serum group. Bars represent mean quantitative amount of each particular MMP [pg/ml] + SEM. ** *p* = 0.003

MMP7 displayed a significantly higher expression in the sera specimen of colon cancer patients in comparison with healthy control serum group (*p* = 0.003) (Fig. 1a). Similarly, we observed a higher abundance of MMP10 (*p* = 0.128) (Fig. 1b) and MMP12 (*p* = 0.544) (Fig. 1c) in colon cancer patients` sera as compared to serum specimens of healthy individuals.

The analysis of MMP7, MMP10 and MMP12 expression regarding clinicopathological characteristics is shown in Table 2. For MMP7 we could observe significant differences in the expression level regarding gender (*p* = 0.049), age (*p* = 0.013) and tumor site (*p* < 0.001). MMP10 displayed a significant expressional difference compared to patients age (*p* = 0.003). As for MMP7 we could observe a significant difference of MMP12 expressional level compared to patients’ gender (*p* = 0.004). Only for MMP12 we could assess a significantly higher expression in patients with vascular invasion (V1-stage) compared to V0-staged patients (*p* = 0.018) (Table 2). Clinicopathological characteristics of the patients compared to relative serum expression of MMP7, MMP10 and MMP12 (each <2 and ≥2) are shown in Additional file 1.

**Table 2:** Overview of clinical parameters and relative serum expression of MMP7, MMP10 and MMP12

Patient Characteristics	*N*	Median relative serum expression					
		MMP7	*p*	MMP10	*P*	MMP12	*p*
Gender			0.049		0.138		0.004
Male	51	1.21		1.22		1.19	
Female	24	0.97		0.95		0.88	
Age at operation			0.013		0.003		0.253
<median = 64 years	37	0.95		0.95		1.16	
≥ median = 64 years	38	1.2		1.36		1.03	
T-Stage			0.582		0.957		0.481
T1	2	0.86		0.9		0.42	
T2	8	0.92		1.13		1.11	
T3	53	1.08		1.13		1.07	
T4	13	1.24		1.22		1.19	
N-Stage			0.806		0.074		0.301
Node negative	29	1.06		1.22		1.0	
Node positive	46	1.16		0.99		1.14	
M-Stage			0.244		0.603		0.087
M0	55	1.06		1.13		1.0	
M1	23	1.26		1.13		1.19	
L-Stage			0.629		0.085		0.593
L0	61	1.06		1.18		1.06	
L1	14	1.25		0.81		1.14	
V-Stage			0.067		0.321		0.018
V0	69	1.06		1.13		1.06	
V1	6	2.41		1.86		1.32	
Micro satellite stage			0.053		0.845		0.844
MSS	5	0.68		0.76		0.47	
MSI	2	1.16		0.95		1.01	
Tumor site			<0.001*		0.76		0.758
Coecum	17	0.98		1.22		1.16	
Colon ascendens	14	1.11		0.88		0.78	
Right colon flexure	6	1.28		1.32		1.13	
Colon transversum	3	1.37		0.86		1.0	
Colon descendens	5	2.12		2.16		1.29	
Colon sigmoideum	30	0.96		0.99		1.14	

*In ANOVA *p* < 0.001, the post-hoc Tukey’s test showed a significant difference between colon descendens and coecum, colon ascendens and right colon flexure (*p* < 0.001; *p* < 0.001; *p* = 0.007, respectively)

Taken together our results propose that MMP7, MMP10 and MMP12 are increased in sera of colon cancer patients when compared to healthy donors and MMP12 seems to play a role in vascular invasion.

### Impact of MMP expression on overall survival

After having confirmed differences in the expression of MMP7, MMP10 and MMP12 concentrations in colon cancer patients´ serum samples and serum samples of a healthy control group we correlated these results with patients’ overall survival. For this purpose, the quotient of MMP expression in colon cancer patients´ sera versus expression in sera of healthy individuals was calculated, and relative serum expression (serum expression of colon cancer patients/ serum expression of control serum group) of <2 and ≥2 was then correlated with overall survival [21].

**Fig. 2:**
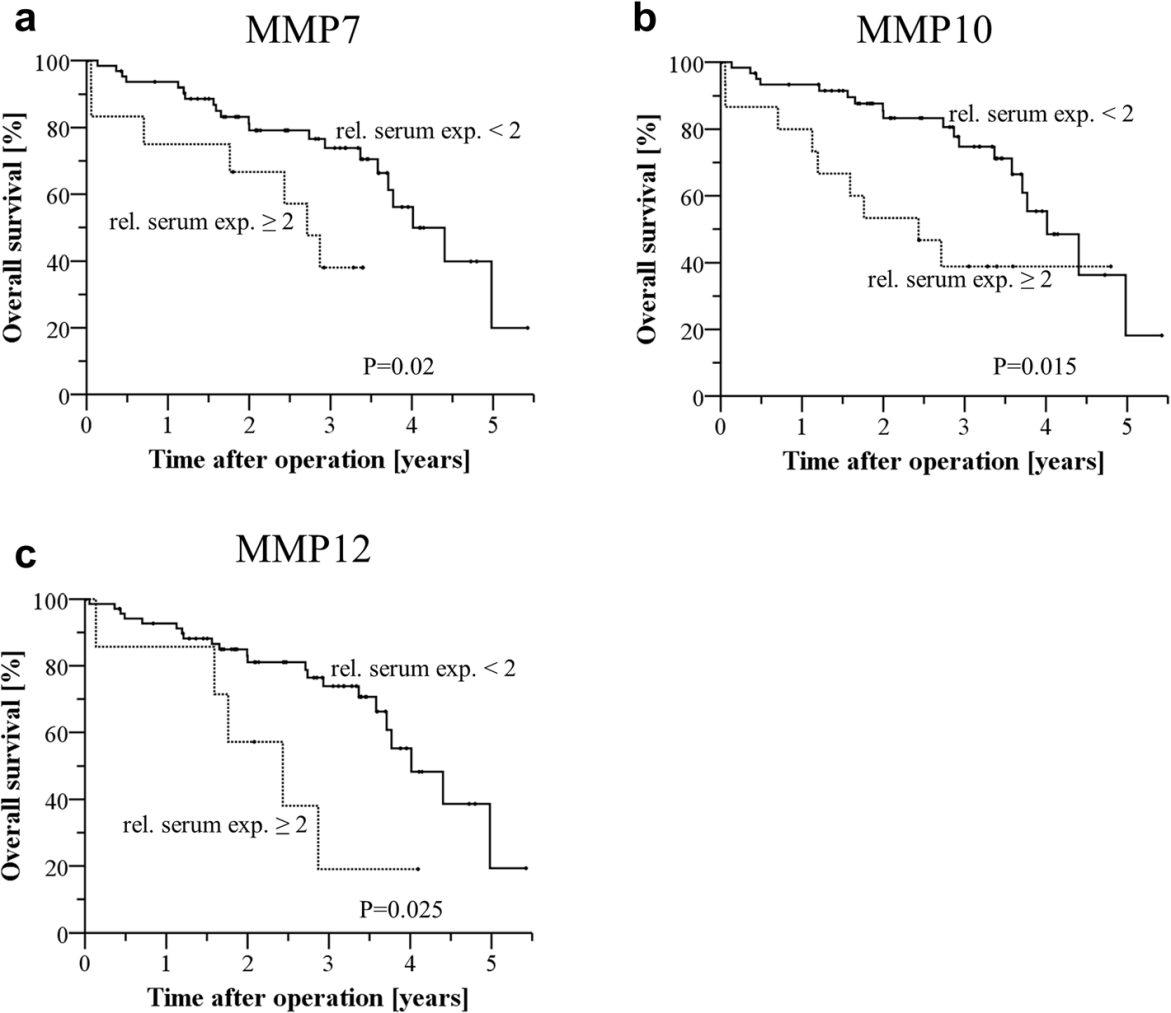
Correlation of relative MMP serum expression (serum/control serum) and overall survival in univariate analysis. An expression ratio serum/control serum ≥2 was defined as increased expression. Overall survival was significantly impaired in patients with increased relative serum expression of (**a**) MMP7 (HR: 2.8 [95 % CI: 1.1–6.8], *p* = 0.02) (**b**) MMP10 (HR: 2.6 [95 % CI: 1.2–5.9], *p* = 0.015) and (**c**) MMP12 (HR: 2.9 [95 % CI: 1.1–7.8], *p* = 0.025). CI = Confidence interval, HR = Hazard ratio

Consistently, a relative serum expression ≥2 (serum expression of colon cancer patients/ serum expression of control serum group) of MMP7 (HR: 2.8 [95 % CI: 1.1–6.8], *p* = 0.02), MMP10 (HR: 2.6 [95 % CI: 1.2–5.9], *p* = 0.015) and MMP12 (HR: 2.9 [95 % CI: 1.1–7.8], *p* = 0.025) correlated with a significantly shortened overall survival (Fig. 2a-c). Strikingly, multivariate analysis revealed that an increased relative serum expression of MMP10 is an independent prognostic marker for impaired overall survival in colon cancer patients (Table 3).

**Table 3:** Multivariate analysis (Cox proportional hazards regression model) of prognostic parameters for overall survival in colon cancer. A MMP10 relative serum expressional ratio ≥2 is an independent prognostic marker in colon cancer patients

Patient characteristics	Hazard ratio	95 % Confidence interval of relative risk	*p*-value
Median age	1.372	0.477–3.943	0.557
Gender	1.275	0.364–4.464	0.704
UICC stage	1.081	0.232–5.027	0.921
T-stage	0.775	0.277–2.174	0.629
N-stage	1.68	0.518–5.447	0.387
M-stage	1.813	0.255–12.912	0.562
R-stage	0.673	0.112–4.033	0.665
Grading	1.127	0.405–3.137	0.820
Adjuvant chemotherapy	1.251	0.236–6.636	0.792
Anastomotic leakage	0.518	0.09–2.992	0.462
MMP7 relative serum expression ≥2	0.901	0.170–4.788	0.903
MMP10 relative serum expression ≥2	3.866	1.069–13.976	0.039
MMP12 relative serum expression ≥2	2.421	0.548–10.695	0.243

## Discussion

The present investigation yielded MMP7, MMP10 and MMP12 serum levels in a homogenous group of colon cancer patients to be adverse prognostic molecular markers exerting valid multiplex bead-based immunoassay - technologies. Strikingly, in multivariate analysis, we could provide evidence that MMP10 is an independent prognostic marker for impaired overall survival. To our knowledge we are the first to demonstrate the influence of serum MMP10 and MMP12 on colon cancer patients´ survival.

MMPs are essential regulators facilitating tumor enhancing processes including tumor growth, migration, invasion, apoptosis, angiogenesis as well as immune-escape mechanisms [10, 22, 23]. Elevated level of several MMPs could be proven in a variety of cancer entities [24–26]. Description of upregulation of several MMPs - among them MMP1,2,3,7,9,10,11,12,13 - in cancerous tissue of colorectal cancer patients is common [27–29]. Moreover for colorectal cancer an association of MMP upregulation with advanced Dukes´stage, more infiltrative phenotype, occurrence of metastatic lesions and unfavorable prognosis was observed [27, 28, 30]. Influxing of MMPs into the bloodstream was verified indicating their role as potential prognostic blood-biomarker [31].

Our results indicate pro-tumorigenic effects of overexpressed MMP7 in colon cancer patients´ sera compared to serum samples of a healthy control group resulting in significantly adverse overall survival. Recent studies have shown that MMP7 is overexpressed in colorectal cancer tissue in comparison to normal mucosa [31, 32]. Furthermore, high expression of MMP7 in colorectal cancer samples exhibits tumor promoting roles in terms of correlation with higher Dukes´stage, lymph node positivity, poor differentiation and metastases. Besides, impaired overall survival appears in patients with colorectal tumors expressing high levels of MMP7 [33, 34]. In consistence with our results, increased serum levels of MMP7 and correlation to worse overall survival in colorectal cancer has been reported [20, 31]. In addition to this, correlation of elevated serum MMP7 with advanced Dukes´ stage and prediction of recurrence in colorectal cancer patients was assessed reflecting higher MMP7 release through tumoral cells due to a higher tumor load [31, 35]. Oncogenic functions of MMP7 rely on different interactions between tumor cells, tumor microenvironment and immune system [20]. In contrast to other MMPs, MMP7 is mainly secreted by cancer cells and activated through cytokines or other MMPs, which are released by tumor stromal cells. This intercellular crosstalk between cancer cells and the tumor microenvironment results in increased tumor growth and invasion [10, 36]. Moreover, MMP7 enhances immune escape mechanisms by blocking lymphocyte cytotoxicity. This immunsuppressive effect is mediated by the cleavage of CD95/CD95, leading to an escape of the immunosurveillance, decreased apoptosis and chemotherapy resistance [20, 37, 38]. These tumor promoting functions of MMP7 are in good accordance with our clinical results and may indicate that a high abundance of serum MMP7 is a surrogate marker for a more aggressive tumor type.

Intriguingly, we were able to show that elevated levels of MMP10 in colon cancer patients´ sera are related to an adverse overall survival by univariate and multivariate analysis. As for MMP7 for MMP10 overexpression in colorectal cancer specimen is described [27, 39]. However, no correlation of elevated tumoral MMP10 level with Dukes´ stage, hepatic metastatic lesions or venous invasion could be proven [27]. Until now, there is no study which reports that overexpression of MMP10 in colon cancer patients´ sera is correlated with survival. Yet, the protumorigenic functions of MMP10 have been well described. Due to stimulation and synergistic effects of other oncogenes like S100A4 (S100 calcium binding protein A4), MMP10 stimulates cell growth and invasion and exerts antiapoptotic properties in vitro [40, 41]. Moreover, in combination with PAI −1 (Plasminogen activator inhibitor) and Plasmin, MMP10 contributes to angiogenesis [42]. These tumor-promoting effects of MMP10 may explain, why high expression of MMP10 in sera of patients with colon cancer is associated with a dismal prognosis. Nevertheless evidence likewise exists that MMP10 also displays tumor-protective capabilities. Koller et al. described an enhanced development of inflammation-associated colonic dysplasia in colitis induced mice lacking MMP10 based on an unremitting inflammation as the major cause. However no changes mediated by MMP10 could be proven in colonic epithelial cells [43]. Due to contradictory effects of MMP10 further investigations are needed to profoundly evaluate the role and differences of MMP10 in colon cancer and inflammatory bowel disease associated dysplasia.

As we have observed for MMP7 and MMP10, we found that elevated abundance of serum MMP12 in colon cancer patients is associated with a significantly shortened overall survival. Consistently with MMP7 and MMP10 also overexpression of MMP12 in colorectal cancer specimen is reported [27]. However divergent evidence exists that - opposing to MMP7 and MMP10 - MMP12 diminishes colon cancer growth in vitro and has a favorable impact on overall survival in colorectal cancer patients [44]. Interestingly, Asano et al. observed higher level of MMP12 in M0-staged patients compared to metastasized patients in primary colorectal cancer [27]. These antitumorigenic effects could be based on MMP12-induced decrease of VEGF (vascular endothelial growth factor) level as well as on an increase of Angiostatin level - both are known to play a crucial role in tumor neovascularization [45]. Contrary, we observed a higher relative serum expression of MMP12 in patients with vascular invasion (V1-stage) compared to V0-staged patients suggesting a tumor promoting role of serum MMP12. Moreover, patients with single nucleotide polymorphism (SNP) in the promotor region of MMP12 display a higher risk of suffering from disseminated colorectal cancer and for stromal MMP12 expression in colorectal cancer liver metastases an association with impaired survival is observed [46, 47]. Moreover invasive and migrative potential of MMP12 is noted in vitro [24]. Therefore MMP12 seems to exhibit both protumorigenic as well as antitumorigenic properties probably due to the fact, if MMP 12 is derived rather from macrophages, stromal or tumor cells and the occurrence of functional single nucleotide polymorphism (SNPs) [44–49]. As for MMP10 until now there are no studies assessing MMP12 expression in colon cancer patients´ sera in regard with overall survival. We suggest a tumor promoting role of MMP12 due to impaired overall survival in colon cancer patients expressing higher serum level of MMP12.

In summary, our results imply a significant influence of serum MMP7, MMP10 and MMP12 in human colon cancer dissemination with considerable impact on overall survival. Moreover we are the first to demonstrate that relative serum expression of MMP10 in colon cancer patients is an independent prognostic determinant for adverse prognosis. Therefore we presume the necessity of measuring serum expression of MMPs to facilitate a prediction of prognosis. The present study evinces MMP7, MMP10 and MMP12 level in a homogenous collective of colon cancer patients´ sera to have predictive significance and boosts the idea that these markers represent essential prognostic factors and are probable molecular targets for tailored, individualized therapeutic approaches for prospective antitumoral strategies.

## Conclusion

Upregulation of several MMPs plays a pivotal role regarding tumor progression, metastatic formation and patients´ outcome likewise in colon cancer. Large studies correlating differential MMP expression in colon cancer patients´ sera are scarce. In the present study we investigated the expression of MMP7, MMP10 and MMP12 in serum specimens in a homogenous collective of colon cancer patients with regard to clinicopathological characteristics and patients´ prognosis. MMP7, MMP10 and MMP12 were significantly associated with a dismal prognosis. Moreover, MMP10 is an independent prognostic marker in colon cancer patients. Overexpression of MMP12 was observed more frequently in patients with vascular invasion. The findings are promising and evince the potential of serum MMP7, MMP10 and MMP12 expression as prognostic biomarker. The results may help to stratify patients into different risk groups. Therefore, further prospective studies with a larger cohort of patients are required.

## Abbreviations

CD95, cluster of differentiation 95; CI, confidence interval; CRC, colorectal cancer; HR, hazard ratio; LEF-1, lymphoid enhancer binding factor-1; M1, metastatic disease; MMP, matrixmetalloproteinases; PAI-1, plasminogen activator inhibitor; RECK, reversion-inducing cysteine-rich protein with Kazal motifs; S100A4, S100 calcium binding protein A4; SEM, standard error of the mean; SNP, single nucleotide polymorphism; TIMP, tissue inhibitors of matrixmetalloproteinases; TNM, tumor node metastases; UICC, union internationale contre le cancer; V1, vascular invasion; VEGF, vascular endothelial growth factor

## Additional file

Additional file 1: Correlation of relative serum expression of MMP7, MMP10 and MMP12 regarding clinical parameters. (DOCX 14 kb)
