# Supplementary material for: Serum MMP7, MMP10 and MMP12 level as negative prognostic markers in colon cancer patients
Source: BMC Cancer. 2016 Jul 18;16:494. doi: 10.1186/s12885-016-2515-7 (PMC4950722; doi:10.1186/s12885-016-2515-7)
Supplement: Additional file 1: — Correlation of relative serum expression of MMP7, MMP10 and MMP12 regarding clinical parameters. (DOCX 14 kb) [file 12885_2016_2515_MOESM1_ESM.docx]

**Additional file 1:** Correlation of relative serum expression of MMP7, MMP10 and MMP12 regarding clinical parameters

| **Patient Characteristics** (number of patients) | MMP7 relative serum expression ≥2 | MMP7 relative serum expression <2 | p-value | MMP10 relative serum expression ≥2 | MMP10 relative serum expression <2 | p-value | MMP12 relative serum expression ≥2 | MMP12 relative serum expression <2 | p-value |
| --- | --- | --- | --- | --- | --- | --- | --- | --- | --- |
| **Gender**  Male  Female | 11 (14.5%)  1 (1.3%) | 41 (53.9%)  23 (30.3%) | 0.09 | 11 (14.5%)  4 (5.3%) | 20 (26.3%)  41 (53.9%) | 0.763 | 7 (9.1%)  0 (0.0%) | 46 (59.7%)  24 (31.2%) | 0.092 |
| **Anastomotic leakage**  Yes  No | 2 (2.6%)  10 (13.2%) | 5 (6.6%)  59 (77.6%) | 0.304 | 2 (2.6%)  13 (17.1%) | 5 (6.6.%)  56 (73.7%) | 0.619 | 0 (0.0%)  7 (9.1%) | 7 (9.1%)  63 (81.8%) | 1.00 |
| **T-Stage**  1 / 2  3 / 4 | 0 (0.0%)  12 (15.8) | 10 (13.1%)  54 (71.1%) | 0.142 | 2 (2.6%)  13 (17.1%) | 8 (10.6%)  53 (69.7%) | 0.982 | 2 (2.6%)  5 (6.5%) | 8 (10.4%)  62 (80.5%) | 0.198 |
| **N-Stage**  negative  positive | 4 (5.3%)  8(10.5%) | 25 (32.9%)  39 (51.4%) | 0.708 | 9 (11.8%)  6 (7.9%) | 20 (26.4%)  41 (53.9%) | 0.052 | 2 (2.6%)  5 (6.5%) | 28 (36.4%)  42 (54.5%) | 0.554 |
| **M-Stage**  0  1 | 6 (7.9%)  6 (7.9%) | 47 (61.8%)  17 (22.4%) | 0.168 | 11 (14.5%)  4 (5.3%) | 42 (55.3%)  19 (25.0%) | 1.00 | 4 (5.2%)  3 (3.9%) | 50 (64.9%)  20 (26.0%) | 0.42 |
| **Resection margin status**  0  1 | 10 (13.9%)  1 (1.4%) | 58 (80.6%)  3 (4.2%) | 0.493 | 13 (18.1%)  1 (1.4%) | 55 (76.4%)  3 (4.2%) | 1.00 | 6 (8.3%)  1 (1.4%) | 62 (86.1%)  3 (4.2%) | 0.342 |
| **Grading**  2  3 | 10 (13.3%)  2 (2.7%) | 43 (57.3%)  20 (26.7%) | 0.491 | 13 (17.3%)  2 (2.7%) | 40 (53.3%)  20 (26.7%) | 0.205 | 5 (6.6%)  2 (2.6%) | 48 (63.2%)  21 (27.6%) | 1.00 |
| **Occurrence of liver metastases**  Liver metastases (syn-& metachronous)  No liver metastases | 6 (7.9%)  6 (7.9%) | 19 (25.0%)  45 (59.2%) | 0.193 | 5 (6.6%)  10 (13.2%) | 20 (26.3%)  41 (53.9%) | 1.00 | 3 (3.9%)  4 (5.2%) | 23 (29.9%)  47 (61.0%) | 0.683 |
| **Adjuvant Chemotherapy**  Yes  No | 6 (7.9%)  6 (7.9%) | 40 (52.6%)  24 (31.6%) | 0.524 | 6 (7.9%)  9 (11.8%) | 40 (52.6%)  21 (27.6%) | 0.084 | 4 (5.2%)  3 (3.9%) | 43 (55.8%)  27 (35.1%) | 1.00 |
| **Survival**  Dead  Alive | 7 (9.2%)  5 (6.6%) | 21 (27.6%)  43 (56.6%) | 0.112 | 9 (11.8%)  6 (7.9%) | 19 (25.0%)  42 (55.3%) | 0.07 | 5 (6.5%)  2 (2.6%) | 22 (28.6%)  48 (62.3%) | **0.048** |
